# The discharge conversation: a phenomenographic interview study

**DOI:** 10.1186/s12912-020-00452-8

**Published:** 2020-07-01

**Authors:** Kyriakos Theodoridis, Adina Noghi, Gunilla Borglin

**Affiliations:** 1 Department of Care Science, Faculty of Health and Society, Malmö University, SE-20506 Malmö, Sweden; 2 Department of Surgery, Skåne University Hospital, Malmö, Sweden; 3 Department of Nursing Education, Lovisenberg Diaconal University College, 0456 Oslo, Norway

**Keywords:** Information, Communication, Nurse-patient-relationship, Discharge conversation

## Abstract

**Background:**

Studies have highlighted deficiencies in the information given by nurses to surgical patients. Studies also show that the role of the nurse in connection with the discharge of patients after surgery is unclear. The aim of the study was therefore to elicit and to explore registered nurses’ conceptions of the phenomenon of *nursing care information given to surgical patients in connection with hospital discharge*.

**Method:**

Semi-structured interviews were conducted with fifteen nurses at surgical unites at the southern parts of Sweden. The interviews were transcribed and then analysed according to the phenomenographic approach.

**Result:**

The analysis resulted into three descriptive categories which conjointly may be said to express the general conceptions of the informants. Thus, according to the informants, the provision of nursing care information in connection with the discharge of surgical patients is: (i) not a nursing priority, (ii) adapted to the context of care, and (iii) a possible enhancement of the nursing process and the quality of care.

**Conclusion:**

The result of the study implies that the discharge conversation may be seen as an opportunity for the nursing profession to formalise and to enhance the quality of care in connection with the discharge of surgical patients.

## Background

This study is concerned with the discharge process of surgical patients and with the fact that the role of the nurse within the discharge process is unclear. While the topic has not been the focus of systematic research, studies in Japan and the USA make evident that the responsibilities of the nurse in connection with the discharge process diverge substantially from place to place [1–7]. In regard to complexity of post-surgical self-care, it is generally acknowledged that discharge planning and patient teaching is of crucial importance in order to secure optimal rehabilitation and to prevent avoidable readmissions to the hospital [8–11]. On this ground, since the role of the nurse with respect to the discharge of patients after surgery seems to be unclear, the topic deserves closer scrutiny.

In order to frame the process of patient discharge, two notions will be employed which derive from the Swedish professional healthcare nomenclature, namely, ‘admission conversation’ (Swedish ‘inskrivningssamtal’; i.e. ‘writing-in-conversation’) and ‘discharge conversation’ (Swedish ‘utskrivningssamtal’; i.e. ‘writing-out-conversation’). Although these notions do not belong to the standard terminology associated with the nursing process, they will be employed in order to denote the formal meeting sessions held by the physician (and occasionally by the nurse) with the patient when the hospitalisation period of the patient begins (and the patient is admitted to the hospital for surgery) and when the hospitalisation period comes to an end and the patient is about to go home.

If the *admission conversation* occurs at the beginning of the hospitalisation period, the *discharge conversation* may be said to mark the end point of hospitalisation when the obligations and the responsibilities of the health team with respect to the patient may be said to cease. As remarked in a recent study of medical nurses’ discharge planning at Jamaican hospitals, ‘Within clinical settings, the transition process for successful discharge planning activities and health education for clients should commence on the first day of admission’ [5]. On this ground, the admission conversation and the discharge conversation should be considered to offer two auspicious opportunities for the nurse to communicate with the patient in regard to surgery, medication, self-care and other issues of relevance.

The notions of communication and conversation are connected, of course. Although the importance of sound communication strategies cannot be overestimated within contemporary nursing, nonetheless, communication may fail if nurses misconceive *why* and *when* communication should take place. This is particularly evident in connection with surgery where wise and efficient communication proves essential for the transition of surgical patients from nursing care and hospitalisation to self-care and autonomy at home [12, 13]. In particular, to be able to handle the daily activities after discharge, patients must have knowledge of several issues including the specific healing process in order to prevent possible complications [9].

As is highlighted by many studies, the lack of adequate information may often yield negative patient experiences, including feelings of frustration, helplessness and unsafety at home [1, 2, 8]. In addition, inadequate communication strategies may result in repeated and unnecessary contacts with health care deliverers in order to gain information which should have been given to the patients in connection with the discharge after surgery [7, 12, 14].

Generally, it is well-known that peri-operational communication encompasses various types of information which in the optimal case should result in a personalized set of instructions regarding nutrition, wound and pain management, medication and rehabilitation [8, 9, 14]. Thus, various kinds of information need to be custom-made in order to facilitate the discharge of the patient. Since proper self-care after discharge is essential to the recovery of the patient, and since receiving correct information is essential to proper self-care, it seems crucial that the nurse establishes wise and efficient communication strategies in regard to the patient in regard to the discharge from hospitalisation after surgery [1, 12, 13, 15].

The possibility of prudent and efficient communication with the patient is crucial also with respect to the safety dimension of nursing practice. We could say that if communication fails, optimal safety is more likely to fail. Conversely, if communication is successful, the probability of promoting optimal safety increases [12]. For this reason, a correct understanding of the possibility of the discharge conversation with patients after surgery seems to be an important feature of the nursing responsibility for healthcare safety.

Communication is also considered of fundamental importance to the successful implementation of the *nursing process*, commonly delineated according to the phases of *assessing*, *planning*, *implementing* and *evaluating* nursing interventions [16]. It may be observed that each phase of the nursing process corresponds to various kinds of communication which as such may involve all levels of linguistic activity (spoken, written, non-verbal). Thus, if nurses aim to implement the nursing process in a constructive and efficient way, the type of communication pertaining to the various phases of the nursing process should be made salient and reflected upon. In connection with surgery, this means that the discharge conversation, considered from the vantage point of the practicing nurse, denotes an opportunity to communicate essential information to the surgical patient before home-going. On this grounds, it seems pertinent to study how nurses conceive the issue of communication in regard to patient discharge after surgery. The aim of the study was to elicit and to explore the informants’ conceptions of the phenomenon of *nursing care information given to surgical patients in connection with hospital discharge*.

## Method

In order to realise the aim, we conducted interviews that were recorded, transcribed and then analysed in accordance with phenomenography. Phenomenography is an empirical approach employed to investigate different ways of understanding a certain phenomenon. Such ways are also called *conceptions* and are commonly presented in terms of *descriptive categories* [17–19]. Phenomenography was considered particularly relevant since the study aimed to explore and delineate various conceptions of a certain phenomenon.

Regarding *phenomenography*, the question may arise whether the correct term is ‘conceptions’ or ‘perceptions’. Merton states that ‘conceptions’ and ‘ways of understanding’ should not be understood as subjective qualities but rather as ‘conceptions of reality’ or ‘categories of description’ which may be used in the understanding of ‘concrete cases of human functioning’ [17]. This view depends on the distinction between the first-order and the second-order perspective which may be exemplified by means of distinguish between (say) the statement that ‘the discharge of patients is a nursing priority’ (which corresponds to the first-order understanding of how things should be in the world), and the statement ‘some people think that the discharge of patients is a nursing priority’ (which corresponds to the second-order understanding of *certain ideas* of how things should be in the world). On this ground, while *perception* denotes the experiential act of pre-reflective awareness of something, the term *conception* is not necessarily tied to the sensory realm of perceiving. For this reason, the notion of *conception* was deemed more appropriate with respect to the present study.

### Study context

The informants recruited for this study worked at surgical care units and were engaged in both planned and acute surgical care. The units, that are located at five different county hospitals in the southern parts of Sweden, may be said to operate standard as well as complex surgical interventions which result in a varied number of patients and in-hospital days before discharge for the patients. The size of each unit varied between 10 to 26 beds. Health care professionals at these units worked in teams mainly consisting of one registered nurse (RN), two health care assistants and one or two physicians. Each team could be responsible for between 2 to 10 patients.

### Recruitment and sample

A convenience sampling technique was used to recruit fifteen RNs working at eight different surgical units. The managers at each surgical ward received written information about the study together with an invitation to participate in the study. The inclusion criteria for participation were to be an RN and to have at least 6 months experience from working at a surgical ward in order to participate in the study. Initially nineteen informants accepted the invitation to participate, however four informants withdraw at a later stage. A total of fifteen informants participated in this study, two men and thirteen women. The informants all held a Bachelor of Science in nursing; four of them were specialist nurses in surgical care. Working experience varied from one to 24 years with an average of 9 years and 4 months of working experience.

### Semi-structured interviews

The interviews were conducted between September and October 2018. The data collection and the study in general were conducted in compliance with the principles of research ethics in accordance with the Declaration of Helsinki [20]. Before the interviews, the participants were informed about the purpose of the study, that their identity was confidential and that they have the right to refuse to participate or withdraw from the trial at any time without questions asked.

A semi-structured interview-guide was developed and its suitability was checked by means of two test-interviews which in essentials confirmed the suitability of the interview guide and were therefore included in the data-collection and the analysis. The authors took care to construe the questions in accordance with the phenomenographic approach which requires the participant to reflect on the subject matter (and not only to report on feelings and happenings).

More specifically, the interview guide contained questions regarding the participants’ conceptions of (i) their nursing experience, (ii) their workplace, (iii) the information communicated to patients, and (iv) the discharge process at their unit. The initial questions (i/ii) aimed at gaining an understanding of the context within which the phenomenon occurred while the third and fourth question focused the way of dealing with the phenomenon (Table 1). In addition, the participants were asked to clarify, to expand on, or to exemplify certain issues that were brought to the fore in the interview process [21]. The interviews were individual and lasted between 22 to 55 min; total record time was 470 min with an average of 31 min for each interview.

**Table 1:** Overview semi-structured interview questions

• Could you please describe how a discharge conversation is conducted?	
• How would you plan your discharge conversation?	
• What are your perceptions about the discharge conversation?	

### Data analysis

The analysis followed in main outline Ference Marton’s description of the phenomenography approach [17–19]. In the first step, the interviews were transcribed and the transcriptions were read in order to gain an overall understanding of the central themes corresponding to the aim of the study. Statements were identified and differentiated according to the conceptions expressed with respect to the questions asked. In the second step, the statements were organised according to the specific ways of conceiving the phenomenon that emerged in the material. In the third and final phase, by means of a dialogue among the research group regarding similarities and differences within the selected statements, three descriptive categories were identified and articulated which constitutes the result of the study.

## Result

The analysis resulted into three descriptive categories which conjointly may be said to express the general conceptions of the informants. Thus, according to the informants, *the provision of nursing care information in connection with the discharge of surgical patients* is:

(I) not a nursing priority
(II) adapted to the context of care
(III) a possible enhancement of the nursing process and the quality of care

The categories may be commented in more detail as follows.

Category I: Is not a nursing priority.

According to the first descriptive category, the informants generally conceived that *the discharge of surgical patients* was not of a nursing priority. Rather, it was considered to belong to the responsibility of the physician. The view emerged that the priority of the nurse was to attend to the care of the patients. Some of the significant voices are the following:

“It is the physician who is responsible for the discharge and we support with information if we think that something is missing.” (RN 6).

“The physicians generally hold the discharge conversation at the ward. … we [the RNs] are not always present. … they [the physicians] talk to the patients themselves … and then we usually cut the tape and take the needles out and ask if they [the patients] have any more questions about the medical information or if they have any questions to us. So it is usually the physicians that take care of it … actually.” (RN 12).

The nursing priority, the informants stated, was to care for the patients.

“The patients in worst condition must be my priority. The fact is that those who are about to get discharged are the healthiest. So if I have five other patients that are not ready to be discharged but have great caring needs, then I spend my time on those patients.” (RN 3).

Moreover, nursing care information was considered to be complementary to medical information in the sense that not all patients needed ‘a nursing discharge conversation’ but only those patients who had experienced certain problems regarding some specific nursing issue.

“I would say it [the physician’s discharge information] includes sufficient medical information as the patients are rarely calling back and asking. Most often it is rather a lack of nursing care related information.” (RN 15).

Thus, on the first descriptive category, the discharge conversation was not considered of primary importance to the provision of nursing care information to surgical patients.

Category II: Nursing care information at discharge – adapted to the context of care.

The second descriptive category reflects the participants’ conceptions of subsidiary factors that may influence the provision of nursing care information, for instance, the actual workload, the time pressure, the prevailing routines, and so on. In addition, the participants stated that nursing care information was communicated continuously during the course of the hospitalisation of the patient. On this view, information was provided at different situations, depending on the specific needs of the patient. Some representative voices state:

“The information is given over the whole hospitalization.” (RN 8).

“It’s important to inform them [the patients] during their whole stay. The more information they get during their stay [at the hospital], the less they ask at the discharge conversation.” (RN 5).

“So … we usually inform meanwhile we take care of our patients, we do not focus it all information in the end. There is no need to repeat information.” (RN 3).

The informants conceived that the provision of nursing care information was not necessarily tied to a formal discharge conversation. In particular, although conversations between the nurse and the patient were considered important, the ‘last’ conversation with the patient was conceived less important than the ‘first’ admission conversation. The participants expressed the worry that ‘too much information’ to the patient might be counterproductive. Some voices state:

“The discharge conversation does not have the same priority as the first admission conversation between patient and nurse.” (RN 7).

“You know, if you are given too much information, it is hard to take in.” (RN 3).

The participants further deemed that the spatial arrangements as well as the structure of work could influence whether a formal nursing discharge conversation took place or not. We read:

“If you are going into a private room and you are allowed to talk and not to lie in your bed with three different persons around you. From the patient’s perspective, it might be much easier to communicate if you are not surrounded by such a constant buzz.” (RN 3).

“It is mostly about a lack of resources. If we had more resources then we could offer more focused time instead of making point efforts and running around like little chickens and then saying goodbye’.” (RN 14).

“Unfortunately, it is often far too heavy workload and too stressful to keep up with the discharge information.” (RN 4).

“I feel a bit sad that we do not are have these discharge conversations together [with the physicians], but I don’t know … , it is usually the physicians doing it by themselves.” (RN 12).

Thus the provision of nursing care information was considered to depend upon various contextual factors such as working routines, team collaboration (or lack thereof), work load, time pressure and the spatial organisation of the ward.

Category III: Nursing discharge conversation: a possible enhancement of the nursing process and the quality of care.

The third category depicts the conception that emerged when the informants were reflecting upon the possibility of a formal nursing discharge conversation with the patient. The participants stated that such a discharge conversation might benefit the patient on several points, for instance, it may provide with an opportunity to summarise the events of the hospitalisation, to discuss and make evident the details regarding the self-care of the patient, to articulate recommendations from the other members of the healthcare team, and in general to ensure that the patient has understood the essential points regarding wound care, nutrition, medication, and rehabilitation. Some representative voices states:

“Ideally for me would have be to have the opportunity and the time I need for an individual meeting with the patient for nursing care discharge information. … We would probably need to structure some form of standardised discharge process. … You should sit down as you do in the admission... Because, at the admission you want a clear picture, you want to have the patient from the beginning … If you do the same at the discharge, putting effort and energy, you would gain a lot and then you could close the caring session with the patient all together as a team with the physician and the nurse and so on.” (RN 4).

“That would be the best … that family and next of kin could be present at the discharge conversation … Because they have a lot of questions. … That they are present at the admission, and at the discharge, then they would get the whole thing.” (RN 5).

“If we could build a group and discuss what nursing care is, what we should inform [the patients] about at a discharge meeting. … I would have wanted some sort of checklist to go through those points.” (RN 6).

The informants emphasised the fact that that patient’s need for information doesn’t stop at discharge from the hospital. Many times it is ‘really important’ to be able to talk with the nurse after having returned home. Perhaps, the informants conjured, certain unnecessary phone calls after discharge would have been prevented if a formal discharge conversation had taken place between the nurse and the patient.

“It could have been good if we had information at discharge from hospital, to close and make a summary the hospitalization.” (RN 8).

“I think it is very important [to have a discharge conversation] … To have a contact when you have come home when all the questions arise … the questions you do not know before your are at home.” (RN 9).

Thus, when thinking about the phenomenon, the informants conceived that a formal discharge conversation might contribute to the realisation of the ideal of person centered care.

### Methodological limitations

As mentioned, the Swedish language that was used in the interviews of this study, contains the expressions ‘inskrivningssamtal’ and ‘utskrivningssamtal’ which may be translated as ‘admission conversation’ and ‘discharge conversation’ respectively. These expressions are generally accepted and employed by healthcare professionals generally. It should be borne in mind that the terms employed express a standard meaning when used within the Swedish healthcare arena.

In English, these expressions are not established in the same way within the healthcare community, as far as we know. For this reason, we have used the notion of ‘first conversation’ which denotes the patient’s ‘admission conversation’ with the physician and the nurse when the patient comes to the hospital for surgery. The notion of ‘discharge conversation’ is meant to denote the ‘last’ conversation of the patient with the physician (and occasionally also with the nurse) before the patient is going home after surgery. These two conversations therefore denote the formal occasions when surgical patients first enter and then leave the hospital.

Evidently, there are limits to the possibility of generalising from the result of a small interview study as this. Nevertheless, it is plausible to assume that the main categories reflect various not uncommon conceptions among nurses. It may be the case that certain surgical wards have established a more rigorous discharge process or even a formal discharge conversation.

The specific context sets limits to the transferability of the result into different contexts, in particular when considering that the notion of ‘surgical patients’ may include a vast range of different kinds of interventions and peri-operational implications. Nevertheless, the result of the present study expresses a valuable point in regard to the discharge of surgical patients, considered from the vantage point of the registered nurse.

## Discussion

This study aimed to elicit and to explore the informants’ conceptions of the phenomenon of *nursing care information given to surgical patients in connection with hospital discharge*. Since the phenomenography approach aims to describe various *conceptions* of a given phenomenon, the present study yielded three descriptive categories which in main lines reflected the conceptions of the informants.

We recall the display of the categories, according to which, *the provision of nursing care information in connection with the discharge of surgical patients* is: (I) not a nursing priority, (II) adapted to the context of care, and (III) a possible enhancement of the nursing process and the quality of care.

According to Category I, the provision of nursing care information to surgical patients in connection with discharge was not considered to be a nursing priority, for some good reasons and perhaps also for some not so good reasons. On a general view, it may be suggested that the surgical wards included in this study entertain two parallel processes of discharge of patients after surgery: one rigid process conducted by the physician and one non-rigid process related to the nurse. According to Category I, nurses prioritised patients in the immediate need of actual caring. On this view, the communication with patients in connection with discharge was not conceived as a priority for the nurse but rather as a priority for the physician. Rather, the priority for the nurse was understood in terms of immediate care (and not in terms of providing with information).

Set against each other were the RNs description of the physicians’ structured and strategical routine for handling relevant medical discharge information to the patients, on the one side, and on the other side, the description of the RNs own none routinized praxis for transferring nursing information in relation to discharge while they simultaneously called for structured discharge information and increased teamwork. The latter is especially important as teamwork has through research been described to improve patient care planning, to be clinically efficient as well as to facilitate a person-centred care [8, 9]. Working in interprofessional teams to ensure continuity of care, patient safety and quality of care is additionally one of the six standards suggested for RNs to possess to meet optimal health care standards [2].

Category II reflects various conditions supporting or hindering the provision of nursing care information to patients in connection with discharge. In particular, this findings gave voice to the well-known report of *time pressure* and *work overload* that characterises contemporary nursing and healthcare at large.

Nurses’ conception of nursing related information was governed by the patients’ needs. The result indicates different conceptions about what the information should contain and when it should be communicated to the patients and their families. In short, different conceptions emerged regarding how to individualize the nursing information to the patients.

Previous studies showed that patients have the expectation in connection with discharge to get a summary history about hospitalization, advice for self-care at home and related nursing care information [3, 9, 10]. Some nurses were uncertain about how the patients understood nursing information which were given at discharge because it was not always individualized to each patient. This finding goes against the well known fact of the importance of finding out what the patients need to know in order to be able to take care of themselves after discharge [4, 13, 22].

Patients desire to be involved in the discharge process but lack of communication could make this wish impossible to fulfil [10]. For instance, patients that have undergone colorectal surgery reported not feeling fully recovered in connection with the discharge [7]. Clinical implications related to this part of the result are that nurses need to exercise critical thinking regarding their communication strategies in order to ensure quality and safety for patients.

Category III, which is perhaps the most telling finding of the study, emphasises the possibilities that the nurses envisaged when they reflected upon the phenomenon and upon the actual routines in connection with the discharge of surgical patients. The outcome of their reflections pointed to the fact that a formal discharge conversation might benefit the patient and also contribute to the realisation of optimal care.

It was claimed that nurses might need to find structure in their communication with the patients in connection with discharge. Some nurses suggested a guide with thematic nursing issues which can be discussed by the nurse and the patient together.

The non-systematic way of dealing with the discharge processes is consistent with previous research that has shown that the patient’s last day at the hospital is experienced as confusing and stressful [7]. A formal discharge conversation might prevent much confusion and misunderstanding.

## Conclusion

The findings of this paper, although not generalizable without qualifications, nevertheless suggest that the possibility of a formal nursing discharge conversation in connection with surgical patients deserves further consideration by nurse researchers as well as practitioners.

## Data Availability

The datasets used and/or analysed during the current study are available from the corresponding author on reasonable request.
